# Genetic Diversity and Natural Selection of the *Plasmodium knowlesi* Circumsporozoite Protein Nonrepeat Regions

**DOI:** 10.1371/journal.pone.0137734

**Published:** 2015-09-17

**Authors:** Mun Yik Fong, Md Atique Ahmed, Shen Siang Wong, Yee Ling Lau, Frankie Sitam

**Affiliations:** 1 Department of Parasitology, Faculty of Medicine, University of Malaya, Kuala Lumpur, Malaysia; 2 Department of Wildlife and National Parks Peninsular Malaysia, Kuala Lumpur, Malaysia; Centro de Pesquisa Rene Rachou/Fundação Oswaldo Cruz (Fiocruz-Minas), BRAZIL

## Abstract

**Background:**

*Plasmodium knowlesi* is a simian malaria parasite that has been identified to cause malaria in humans. To date, several thousand cases of human *knowlesi* malaria have been reported around Southeast Asia. Thus far, there is no detailed study on genetic diversity and natural selection of *P*. *knowlesi* circumsporozoite protein (CSP), a prominent surface antigen on the sporozoite of the parasite. In the present study, the genetic diversity and natural selection acting on the nonrepeat regions of the gene encoding *P*. *knowlesi* CSP were investigated, focusing on the T-cell epitope regions at the C-terminal of the protein.

**Methods:**

Blood samples from 32 knowlesi malaria patients and 2 wild monkeys (*Macaca fascicularis*) were used. The CSP of the *P*. *knowlesi* isolates was amplified by PCR, cloned into *Escherichia coli*, and sequenced. The nonrepeat regions of the CSP gene were analysed for genetic diversity, natural selection and haplotypic grouping using MEGA5 and DnaSP version 5.10.00 programmes. A haplotype network was constructed based on the C-terminal (Th2R/Th3R) T-cell epitope regions using the Median-Joining method in the NETWORK version 4.6.1.2 programme. Previously published sequences from other regions (Malaysia Borneo, Singapore) were also included in the analysis.

**Results:**

A total of 123 *P*. *knowlesi* CSP sequences were analysed. Multiple sequence alignment revealed 58 amino acid changes, and 42 novel amino acid haplotypes were identified. Polymorphism was higher in the C-terminal Th2R/Th3R epitope (π = 0.0293, n = 123) region compared to the overall combined nonrepeat regions (π = 0.0120, n = 123). Negative natural selection was observed within the nonrepeat regions of the CSP gene. Within the C-terminal Th2R/Th3R epitope regions, there was evidence of slight positive selection. Based on haplotype network analysis of the Th2R/Th3R regions, five abundant haplotypes were identified. Sharing of haplotypes between humans and macaques were observed.

**Conclusion:**

This study contributes to the understanding of the type and distribution of naturally occurring polymorphism in the *P*. *knowlesi* CSP gene. This study also provides a measurement of the genetic diversity of *P*. *knowlesi* and identifies the predominant haplotypes within Malaysia based on the C-terminal Th2R/Th3R regions.

## Introduction

Human malaria is a disease caused by five *Plasmodium* species namely, *Plasmodium falciparum*, *P*. *vivax*, *P*. *malariae*, *P*. *ovale* and *P*. *knowlesi*. Approximately 219 million cases of malaria infection and 660,000 deaths caused by malaria were reported by the World Health Organization (WHO) in 2010. *Plasmodium falciparum* causes the highest malarial death worldwide followed by *P*. *vivax* [1, 2].

Prior to 2004, malaria infection caused by the simian malaria parasite, *P*. *knowlesi*, was considered rare. However, a large focus of human *P*. *knowlesi* infection was reported in Kapit Division of Sarawak, Malaysian Borneo [3]. Studies conducted later in other parts of Malaysia, Thailand, Singapore, Indonesia, Philippines, Vietnam, Cambodia, and the Indian islands of Andaman and Nicobar also reported cases of *P*. *knowlesi* infection in local human populations [4–11]. Increasing number of cases of *P*. *knowlesi* infection in humans has raised concern for malaria control and elimination, and this parasite has now been recognized as an emerging pathogen [12, 13]. High parasitaemia in human *P*. *knowlesi* infections is associated with disease severity that involves renal failure and liver dysfunction and respiratory distress [14]. Furthermore, a recent study showed that polymorphisms within the merozoite invasion genes (*nbpxa* and *nbpxb*) of *P*. *knowlesi* were linked to hyperparasitaemia and disease severity in human infections [15].

**Fig 1:**
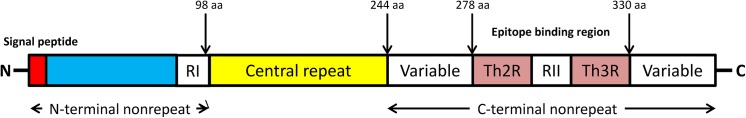
Structure of *P*. *knowlesi* CSP. The repeat region and the C-terminal epitope binding regions Th2R/ Th3R are based on the orthologous *P*. *falciparum* CSP. The amino acid numbering is based on the sequence of *P*. *knowlesi* strain H (GenBank Accession No. XM_002258966.1)

Genes encoding antigens of *Plasmodium* parasites are usually polymorphic and appear to be maintained by selective pressures exerted via host protective immune responses [16, 17]. Evidence for positive selection has been reported for the *Plasmodium* surface proteins; Duffy-binding protein (DBP), circumsporozoite protein (CSP), erythrocyte-binding antigen 175 (EBA-175) and a large number of other antigens [18]. The CSP is one of the prominent surface antigens on the sporozoite of the malaria parasite. The protein is involved in the motility and invasion of the sporozoite from the mosquito into human blood circulation and then to the hepatocytes in the liver. The deduced CSP gene (Fig 1) shows unique features such as two nonrepeat end regions (N- and C-terminals) that sandwich a central repeat region [19, 20]. A five amino acid sequence called Region I (RI), is located immediately upstream of the repeats, and a known cell-adhesive sequence with similarity to the type I repeat of thrombospondin called Region II (RII), is found downstream of the repeat region. In addition, the C-terminal region contains two sub-regions called Th2R and Th3R which are identified as T cell epitope regions [21]. These two regions are polymorphic in natural parasite populations [22]. The CSP gene has been used as marker for genetic polymorphism and natural selection of *P*. *falciparum* and *P*. *vivax* [23, 24]. However, no such studies have been conducted on the CSP gene of *P*. *knowlesi*. Information from such studies can contribute to the understanding of parasite transmission and antigenic variation of the parasite.

Unlike the central repeat region of *P*. *falciparum* and *P*. *vivax* (which contains basically one and two repeat units, respectively), the central repeat region of *P*. *knowlesi* is hyperpolymorphic, with the existence of more than 46 different repeat units arranged in various combinations and lengths [25]. This makes multiple sequence alignment extremely difficult, and meaningful interpretation from such alignment is almost impossible. Therefore, in this present study, the nonrepeat regions of the CSP gene of *P*. *knowlesi* isolates were investigated, with an emphasis on the T cell Th2R/Th3R epitope regions.

## Materials and Methods

### Ethics statement

Ethical clearance for this study was obtained from University of Malaya Medical Ethics Committee (Ref No. 817.18), and University of Malaya Institutional Animal Care and Use Committee (Ref No. PAR/19/02/2013/AA).

### Blood sample collection

A total of 32 blood samples were collected from patients infected with *P*. *knowlesi* from the University of Malaya Medical Centre (UMMC), Kuala Lumpur, Peninsular Malaysia between July 2008 and July 2013. The blood samples were collected by trained nurses in the infectious disease ward of UMMC. All these patients exhibited clinical symptoms associated with malaria. Thin and thick blood smear were prepared from the patient’s blood for microscopic confirmation. Further diagnostic confirmation was done using nested polymerase chain reaction (PCR) and BinaxNOW® malaria rapid diagnostic test. Treatment was administered to patients tested positive for malaria, based on the guidelines of the Ministry of Health, Malaysia. Samples were selected at random with the only selection criterion being that they were only single infection by *P*. *knowlesi*. Ethical approval for this study was obtained from the University of Malaya Medical Centre Ethic Committee (MEC Ref. No. 817.18) and informed verbal consent from the patient was obtained for use of these samples for diagnosis and research. Written consent was found to be unnecessary as verbal consent would be sufficient for the purpose of this study and patient details were noted down for personal recordkeeping. This consent procedure was approved by the University of Malaya Medical Centre Ethic Committee.

Two blood samples were obtained from *P*. *knowlesi* infected monkeys caught by the Department of Wildlife and Nature Parks (DWNP) Malaysia, a government agency which is given the authority to manage and preserve wildlife in the country. The monkeys were captured using monkey traps set up by officers of the DWNP. The monkeys were relocated to a nearby jungle after their blood samples were taken by the officers. No monkeys were sacrificed in this study. The blood samples were tested for *P*. *knowlesi* by microscopy and nested PCR. Ethical approval for the use of monkey blood samples was obtained from University of Malaya Institutional Animal Care and Use Committee (Ref No. PAR/19/02/2013/AA). The approval included sampling techniques used in this study.

### Genomic DNA extraction and nested PCR

Genomic DNA was extracted from 100 μl blood sample by using QIAGEN blood and tissue extraction kit (Hilden, Germany) according to the manufacture’s instruction. DNA was analyzed using *Plasmodium* genus and species specific nested PCR assays based on the *Plasmodium* small subunit ribosomal RNA (SSU rRNA) as described previously [3, 26].

### Cloning and sequencing of the *CSP* gene of *Plasmodium knowlesi*

The CSP gene (Fig 1) was amplified with primers *Pk*CSP-F, 5’-TCCTCCACATACTTATATACAAGA-3’; and *Pk*CSP-R, 5’-GTACCGTGGGGGACGCCG-3’ as described previously [3] with slight modification. PCR amplification was carried out in a 20 μl reaction mixture containing 1X PCR buffer, 2 mM MgCl_2_, 0.2 mM of each dNTP, 0.25 mM of each primer, 1 unit of *Taq* DNA polymerase (Promega, Madison, WI, USA), and 4 μl of DNA template. PCR condition was initiated with an initial denaturation of one cycle at 95°C for 5 min, followed by 40 cycles at 94°C for 30 s, 55°C for 1 min, and 72°C for 2 min in a Biorad MyCycler thermal cycler (BioRad, Hercules, CA). A final elongation step at 72°C for 10 minutes was added to the last cycle. The PCR product was analyzed on 1.5% agarose gel stained with SYBR® Safe DNA gel stain (Invitrogen, USA). The expected size of the *CSP* gene was 1.2 kb. The PCR products were purified with QIAquick Gel Extraction Kit (QIAGEN, Hilden, Germany) and ligated into pGEM^®^-T plasmid vector (Promega Corp.,USA). Each ligation mixture was transformed into *Escherichia coli* Top 10 competent cells. Plasmid DNA from clones having the desired DNA fragment was extracted using the Plasmid DNA Miniprep kit (QIAGEN, Hilden, Germany) according to the manufacturer’s instructions. To detect the possibility of multiple haplotypes infecting a patient or monkey, plasmids from 4–6 recombinant clones from each transformation mixture were sequenced. Sequencing was performed by a commercial laboratory using Big Dye Terminator Cycle Sequencing kit (Applied Biosystems, CA, USA). Nucleotide sequences data were deposited in GenBank (Accession No. KF861695 –KF861766).

### DNA sequence polymorphism and haplotype analysis

The CSP sequences were analyzed as described previously [27, 28]. Sequences from the 453 nucleotides that encode the non-repeat N-terminal (first 195 bp of the coding sequence) and C-terminal (the last 261 bp of the coding sequence) regions of the gene were combined and analysed. The last three nucleotides which encoded the stop codon were not included. Sequences were aligned using CLUSTAL-W described in MEGA5 [29].

Measures of polymorphic sites (S), haplotype diversity (Hd), nucleotide diversity (π), the number of segregating sites (S), and average number of pairwise nucleotide differences within the population (K) were calculated using DnaSP version 5.10.00 [30]. The π was also calculated on sliding window of 100 bases, with a step size of 25 bp in order to estimate the step-wise diversity across the sequence. The rates of synonymous (dS) and non-synonymous (dN) mutations were estimated and compared by the Z-test (P < 0.05) in MEGA 5 using the Nei and Gojobori’s method with the Jukes and Cantor (JC) correction [31]. The dN-dS difference test statistics were applied to test the null hypothesis of strict neutrality of this gene. Tajima's D, Li and Fu's F* and D* values were also calculated using DnaSP v 5.10.00 as a measure of the neutral theory of natural selection.

Orthologous T cell epitope regions Th2R/Th3R in the PkCSP gene was determined by aligning against a *P*. *falciparum* CSP gene sequence (GenBank Accession No. HM582036). In order to look at the pattern of diversity across the Th2R/Th3R epitope regions, π was also calculated on sliding window of 10 bp, with a step size of 5 bp. Pattern of selection across the Th2R/Th3R regions were also tested as above. The Median-Joining method in NETWORK v4.6.1.2 program [32] was used to establish the genetic relationship among CSP Th2R/Th3R haplotypes and to determine the most abundant haplotype.

## Results

### Overall genetic and amino acid polymorphism in the nonrepeat N- and C-terminal regions

The *P*. *knowlesi* CSP gene from 32 human and 2 monkey blood samples were successfully amplified, cloned, and sequenced. From these blood samples, a total of 69 human and 3 monkey *P*. *knowlesi* CSP sequences were obtained (GenBank Accession No. KF861695 –KF861766). An additional 51 sequences available in GenBank (Table 1) were also included in the sequence analysis. These additional sequences were of isolates from Peninsular Malaysia, Malaysia Borneo and Singapore.

**Table 1:** *Plasmodium knowlesi* CSP genes from GenBank. N = total number of isolates.

Population	Country/State	N	GenBank accesion number
Peninsular Malaysia	Peninsular Malaysia	7	M11031.1, EU687467.1, EU687468.1, EU687469.1, EU708437.1, EU687470.1
	Singapore	5	JQ219919.1, JQ219908.1, JQ219920.1, JQ219921.1, JQ219901.1
Malaysian Borneo	Sabah	19	JQ619488.1, JQ619489.1, JQ619491.1, JQ619492.1, JQ619495.1, JQ619498.1, JQ619500.1, JQ619501.1, JQ619502.1, JQ619503.1, JQ619507.1, JQ619505.1, JQ619504.1, JQ619506.1, JQ619496.1, JQ619499.1, JQ619497.1, JQ619494.1, JQ619493.1
	Sarawak	20	GU002532.1, GU002531.1, GU002530.1, GU002529.1, GU002471.1, GU002472.1, GU002476.1, GU002477.1, GU002481.1, GU002482.1, GU002505.1, GU002506.1, GU002511.1, GU002512.1, AY327562.2, AY327566.2, AY327564.2, AY327572.2, AY327570.2, AY327571

**Fig 2:**
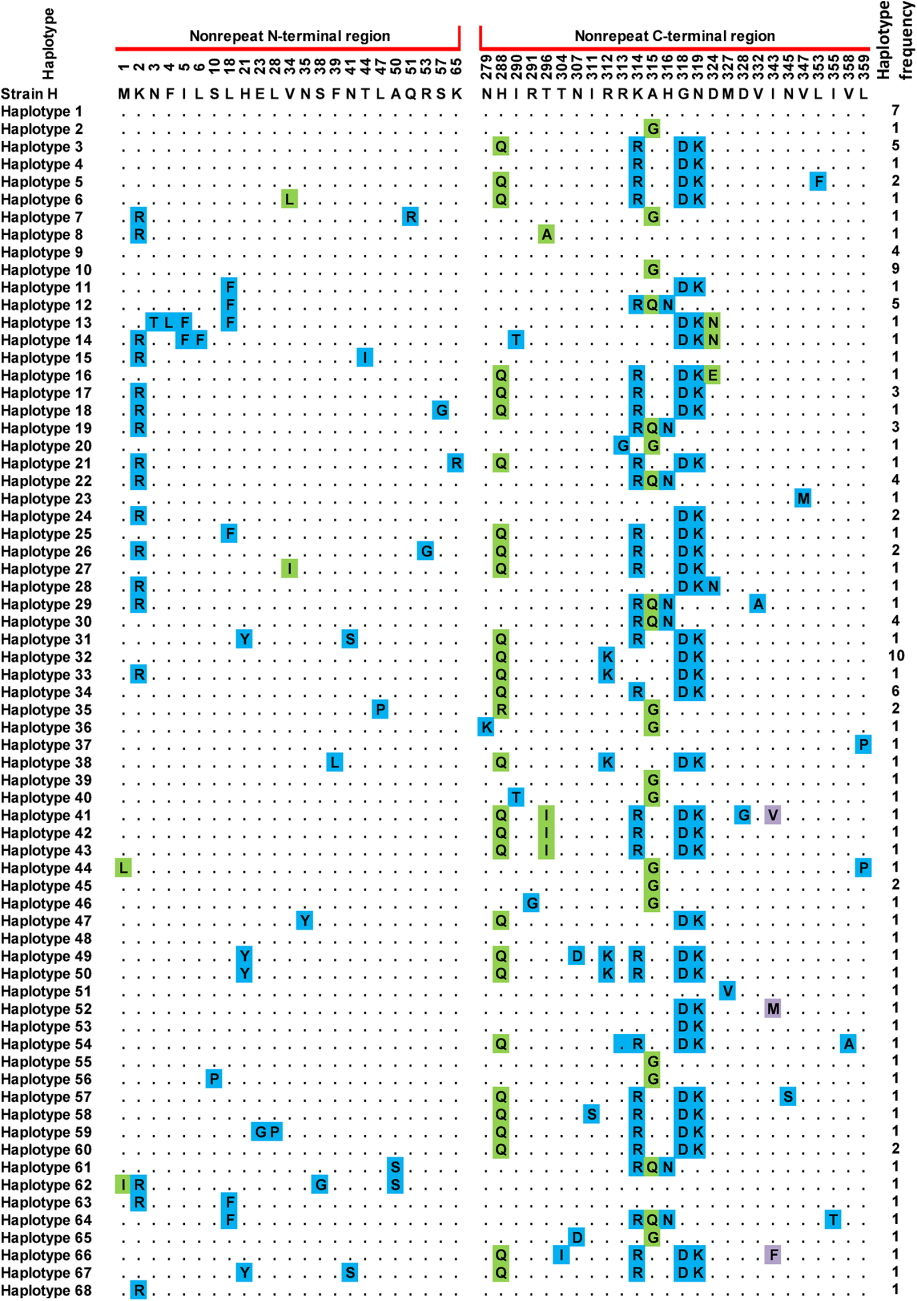
Amino acid sequence polymorphism in the *P*. *knowlesi* CSP N- and C-terminal nonrepeat regions. Polymorphic amino acid residues are listed for each haplotype. Residues identical to those of the reference sequence (strain H) are marked by dots. Monomorphic, dimorphic and trimorphic changes at a particular amino acid position are shaded blue, green and purple, respectively. Total number of sequences for each haplotype is listed in the right panel.

Analysis and comparison of the combined 453 bp nonrepeat region sequences with *P*. *knowlesi* H strain as reference showed point mutations at 83 positions among the 123 sequences. Twenty-six of these mutations occurred at the first base of codon, 23 at the second base and 34 at the third base. Amino acid sequence analysis detected 58 amino acid changes at 49 positions, and 68 haplotypes were identified (Fig 2). The Peninsular Malaysia population showed 54 haplotypes, while the Malaysia Borneo population showed 23 haplotypes. Forty-two of the haplotypes obtained in this study were novel as they were not reported in previous studies.

### Nucleotide diversity and natural selection

**Fig 3:**
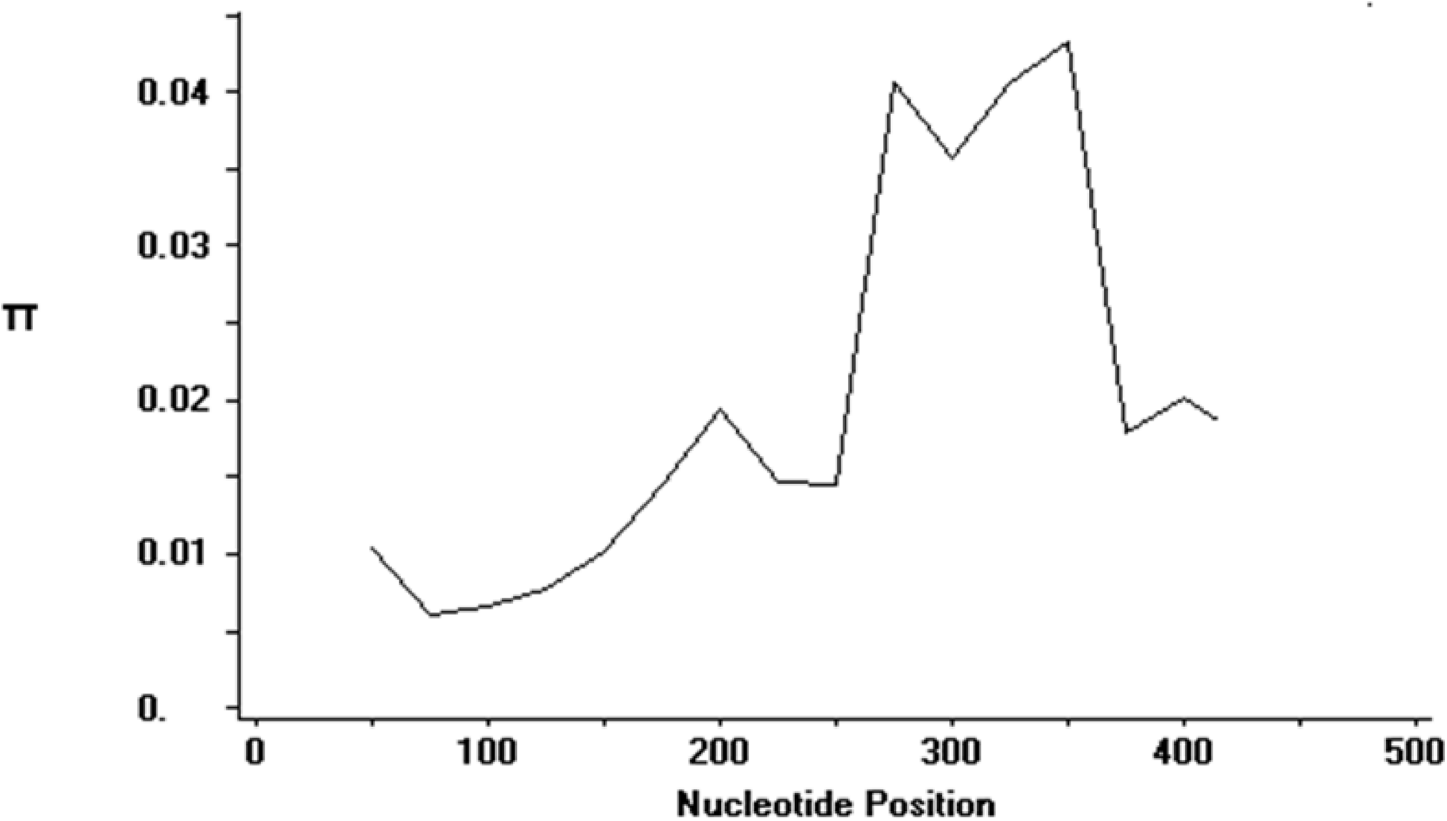
Sliding window plot of nucleotide diversity (π) at the N- and C-terminals of PkCSP. The π values were calculated on DnaSP version 5 with window length of 100 bp and step size of 25 bp.

Apart from amino acid polymorphism, DNA diversity analysis was also conducted on the CSP sequences (Table 2). The average number of pairwise nucleotide differences (K) for the combined 453 bp of nonrepeat regions was 8.5937. Analysis with sliding window plot (window length 100 bp, step size 25 bp) revealed diversity range from 0 to 0.05. Maximum diversity was found between nucleotide positions 250 until 400, which is located in the C-terminal region (Fig 3). The overall haplotype (Hd) and nucleotide diversity (π) were 0.978 ± 0.005 SD and 0.01929 ± 0.00058 SD respectively. To determine whether natural selection contributes to the polymorphism in the nonrepeat regions, the average difference of (dN–dS) was evaluated. The negative value (- 0.023 ± 0.00058 SE) obtained indicates dN < dS. Thus, the nonrepeat regions appeared to be under negative or purifying selection. Tajima’s D statistics was found to be -1.47219 (not significant, P > 0.10). Li and Fu's F* and D* values were found to be -4.78 (p < 0.02) and -4.03 (p > 0.02) respectively (Table 2). Negative values for all these neutrality tests were indicative of a negative natural selection and/or population expansion.

**Table 2:** Estimates of DNA sequence polymorphism and test of neutrality at the nonrepeat terminals and Th2R/Th3R epitope region of *P*. *knowlesi* CSP gene. N = total number of sequences; S = segregating sites; H = number of haplotypes; Hd = haplotype diversity; π = observed average pairwise nucleotide diversity; dN–dS = rate of nonsynonymous mutations minus rate of synonymous mutations,

Gene	N	S	H	Hd ± SD	π ± SD	dN–dS ± SE	Tajima’s D	Fu & Li's D*	Fu & Li's F*
PkCSP	123	83	73	0.978 ± 0.005	0.01929 ± 0.00058	-0.023 ± 0.012	-1.453	-4.78^¥^	-4.03^¥^
PkCSP (Th2R/Th3R)	123	24	27	0.8751± 0.005	0.0293 ± 0.00124	0.004± 0.020	-0.39778	-2.36*	-1.89

^¥^ = p < 0.05

### Sequence diversity in the C-terminal Th2R/Th3R region

**Fig 4:**
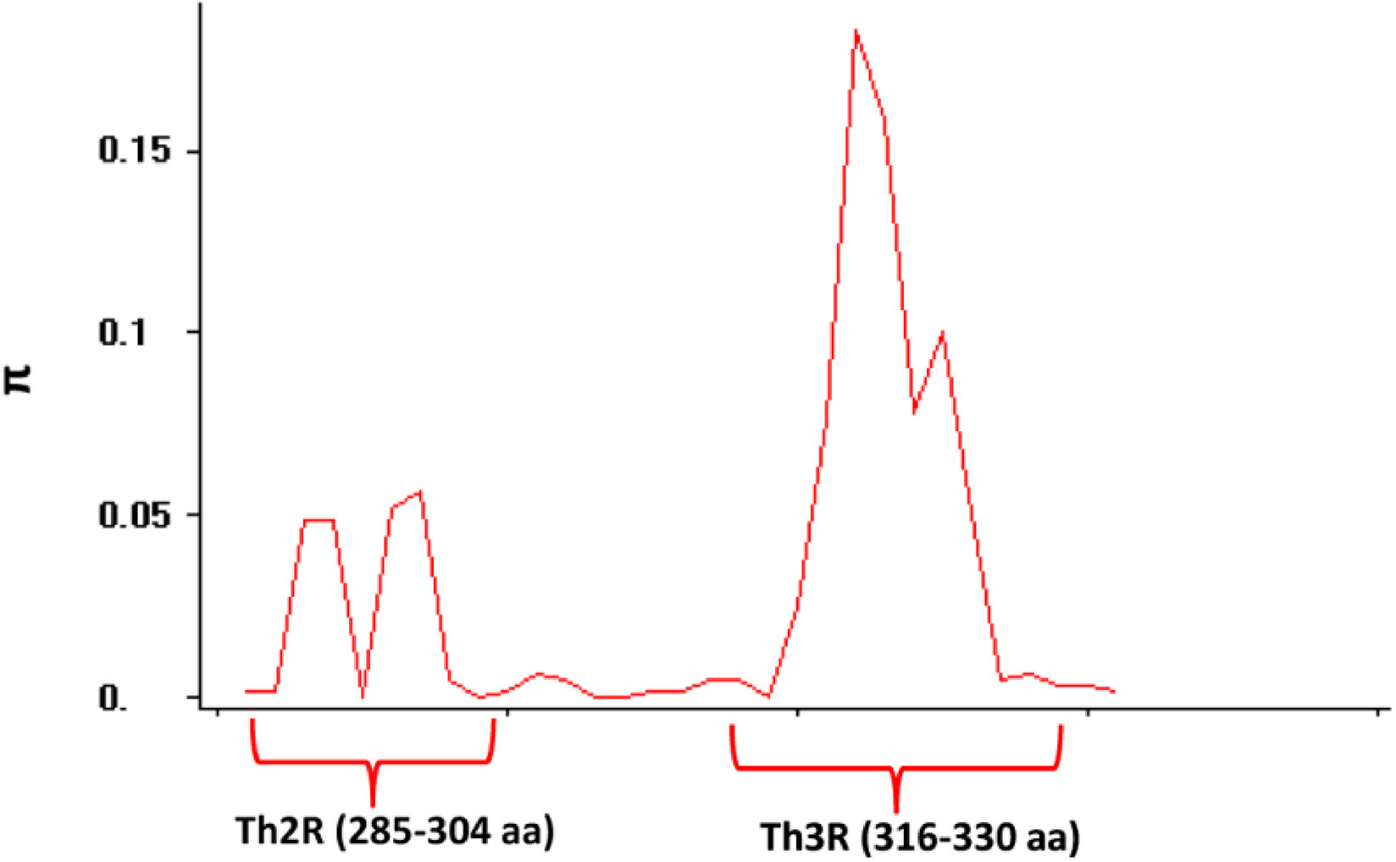
Sliding window (size = 10 bp, length = 5 bp) analysis of genetic diversity (π) across the Th2R/Th3R region. The x-axis represents CSP amino acid positions 278 to 336. The Th2R and Th3R regions are indicated.

The nucleotide diversity (π) within the C terminal Th2R/Th3R epitope region was found to be 0.0293 (Table 2). There were 20 non-synonymous and 5 synonymous substitutions within the region. The sliding window analysis of the polymorphic locus showed that the Th2R/Th3R region contained the highest diversity (Fig 4). The evidence of selection occurring in the regions was not very conclusive as both Tajima’s D statistics (-0.39778, P > 0.10) and Li and Fu's F* and D* values were found to be negative (-2.36, p < 0.05 and -1.89, p < 0.05) (Table 2). However, the dN-dS difference was low positive (0.004 ± 0.020 S.E), which may indicate slight positive selection for this region.

**Fig 5:**
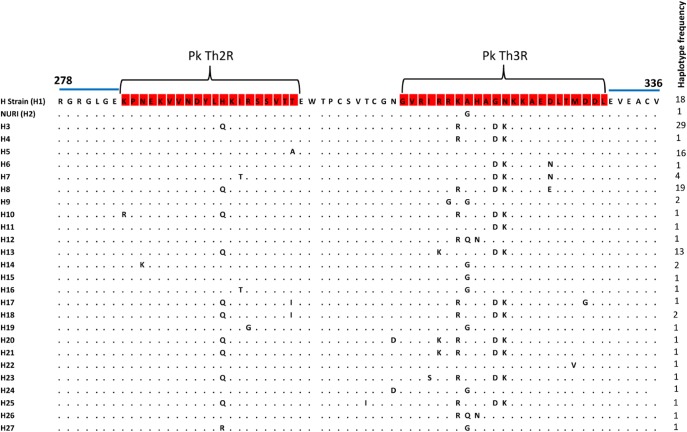
Sequence diversity in the C-terminal nonrepeat region of PkCSP. Sequence alignment showing polymorphism in the nonrepeat C-terminal Th2R/Th3R epitope regions. The highly conserved sequences flanking the Th2R/Th3R domains are indicated by the blue line over the alignment.

**Fig 6:**
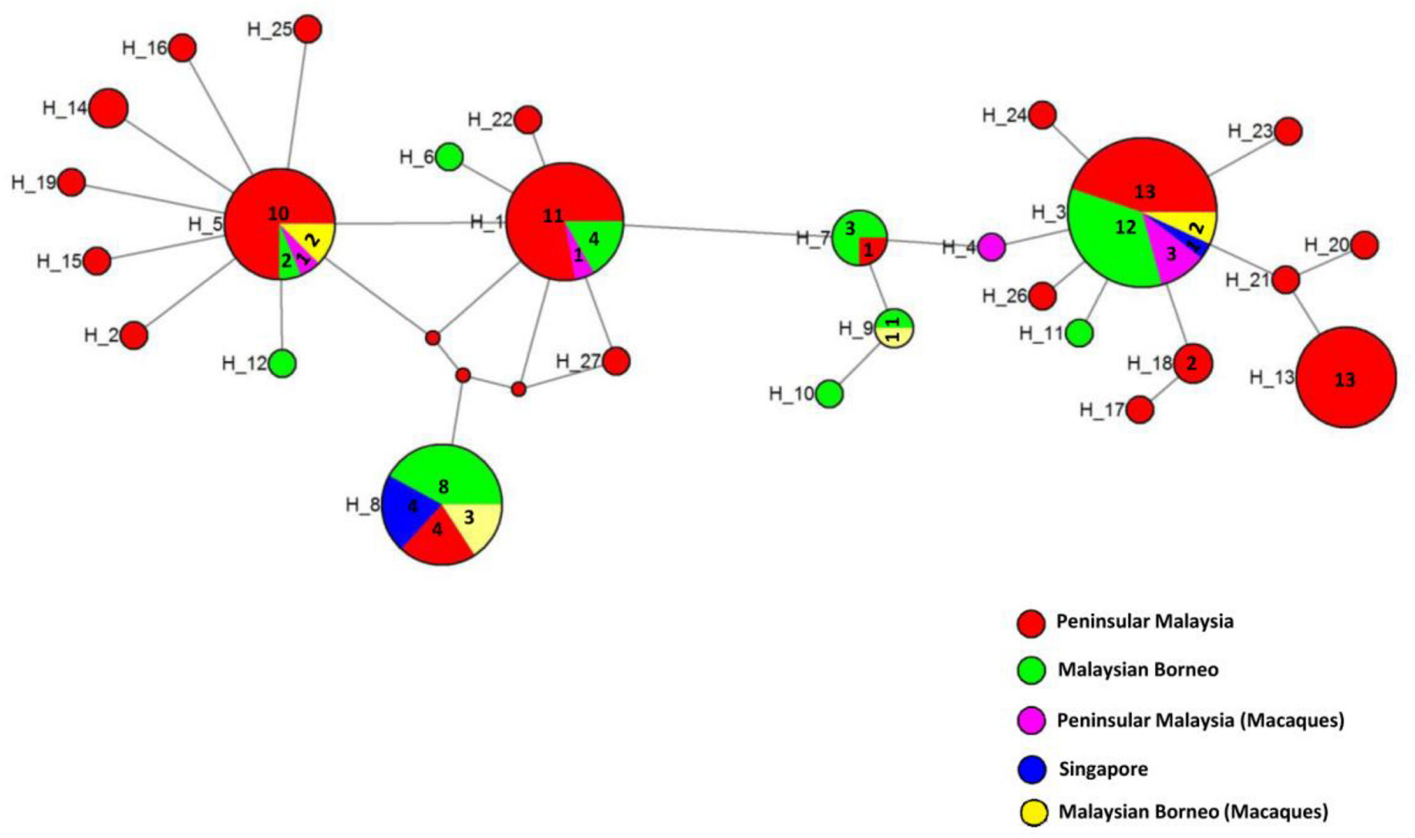
Median Joining Network of CSP generated using Network program. The network shows relationship among the 27 haplotypes based on the human Th2R/Th3R sequences from Peninsular Malaysia, Singapore and macaques from Peninsular Malaysia. Numbers in larger circles represent number of haplotypes, unnumbered circles represent single haplotypes. The smaller red circles are median vectors generated by the program.

A total of 27 Th2R/Th3R sequence haplotypes (Fig 5) were established from the 123 sequences analysed, with haplotype 3 the predominant (n = 29, 23%) followed by haplotype 8 (n = 19, 15%), haplotype 1 (n = 18, 14%), haplotype 5 (n = 16, 13%) and haplotype 13 (n = 13, 10%). The overall haplotype diversity (Hd) for the combined Th2R/Th3R region was found to be 0.8751 ± 0.005 suggesting moderate diversity within the region (Table 2). Haplotype network analysis (Fig 6) also indicated that broadly there were five major haplotypes (haplotypes 3, 8, 1, 5 and 13) with haplotype 3 being most abundant (Table 3). There was no geographical clustering of the haplotypes and many shared haplotypes originating from Peninsular Malaysia and Malaysian Borneo were observed in the Median Joining Network (Fig 6). Four of the abundant haplotypes (i.e. haplotypes 1, 3, 5 and 8) were shared within humans and macaques. Haplotypes from Singapore (haplotypes 3 and 8) were also shared with Peninsular Malaysia and Malaysian Borneo.

**Table 3:** Most abundant PkCSP Th2R/Th2R haplotypes.

Haplotype	n	Amino acid sequence
3	29	QKIRSSVTTEWTPCSVTCGNGVRIRRRAHADK
8	19	HKIRSSVTTEWTPCSVTCGNGVRIRRRQNAGN
1	18	HKIRSSVTTEWTPCSVTCGNGVRIRRKAHAGN
5	16	HKIRSSVTTEWTPCSVTCGNGVRIRRKGHAGN
13	13	QKIRSSVTTEWTPCSVTCGNGVRIKRKAHADK

## Discussion

CSP is the predominant surface antigen of *Plasmodium* sporozoites and is highly immunogenic [33]. Considerable work on genetic diversity and population structure of *Plasmodium* parasites based on the central repeat region has been documented [34]. In the present study, however, the nonrepeat regions was chosen for investigation because the central repeat region is overly diverse due to insertions/deletions during meiotic sexual recombination or intrahelical strand slippage during mitosis [35] and sequence alignment of the repeat region is difficult and uncertain [36].

The full sequence alignment (S1 Fig) obtained in this study shows overall organizational similarity of PkSCP with the CSP of other plasmodia species reported previously, for example PfCSP [37] and PvCSP [38]. The N-terminal nonrepeat region contains the RI with its highly conserved KLKQP core motif adjacent to the central repeat region. The C-terminal nonrepeat region contains the conserved RII and Th2R and The3R epitope regions. RI has been shown to be involved in the binding of CSP to mosquito salivary glands [39, 40]. The upstream residues of RI are also required for binding [39, 40]. Oddly, in PkCSP, the RI upstream region (amino acid positions 66–128) is not conserved (S1 Fig). Substitutions and insertions are found in this upstream region. Most strikingly is the presence of long insertions (KPKQPNVEGDGAKLKQPNEEGDGAKLKQPNEEGDGA; KPKQPNAEGDGAKPKQPNAEGDG) in the CSP of some isolates. Further studies need to be carried out to determine whether these sequence variation affect binding capacity to mosquito salivary glands. The region flanking the C-terminal of the central repeat (amino acid positions 342–393) is hypervariable. Upon closer examination, this region is basically composed of degenerate or partial repeat units of the PkCSP.

Non-synonymous and synonymous mutations are widely used as indicators of the action of natural selection in gene sequences. A significant excess of dN over dS indicates positive natural selection whereas negative values indicate negative or purifying selection [31]. The negative dN-dS value observed for the nonrepeat regions suggests negative or purifying selection. Tajima’s D statistic did not show significant deviation from neutrality which indicates no evidence for diversifying selection. Similar results were also obtained within the Th2R/Th3R epitope regions. Low positive dN-dS value was seen in the Th2R/Th3R regions, which may suggest slight evidence of positive selection. However, this finding should be cautiously interpreted because the dN-dS difference test, unlike the Tajima’s D statistic, is insensitive to demographic factors including population size expansion or contraction. Our results here is similar to the finding of a study on the PfCSP Th2R/Th3R in Madhya Pradesh state of India which reported low dN-dS difference (0.008±0.003) and no evidence of positive natural selection [41].

It has been hypothesized that dominant Th2R/ Th3R haplotypes in different geographical regions are likely to differ, due to ‘mosquito-induced’ selection by the different vector anopheline species transmitting malaria in the respective geographic regions [42]. Studies have shown that Th2R/Th3R sequences in Asia, South America and Africa are geographically distinct with little allele sharing between continents [41, 43]. The Median-Joining network in our study revealed no geographical clustering of the Th2R/Th3R haplotypes. The absence of geographical clustering and occurrence of shared haplotypes between Peninsular Malaysia and Malaysia Borneo *P*. *knowlesi* isolates could be explained in the context of Southeast Asia geo-history. Borneo Island is separated from mainland Peninsular Malaysia by the South China Sea. Geo-historical studies suggest that the separation occurred not so long ago (∼14,000–20,000 years ago). Thus, the *P*. *knowlesi* from Peninsular Malaysia and Malaysia Borneo may be from the same original population which has not diverged significantly over time since the geographical separation. Furthermore, the known mosquito vectors for *P*. *knowlesi* such as *Anopheles cracens*, *An*. *latens*, *An*. *balabacensis* and *An*. *hackeri* are all from the Leucosphyrus Group, whose distribution includes mainland Southeast Asia and Borneo Island [44]. It is likely that the selective pressure exerted by this mosquito group on the PkCSP Th2R/Th3R regions is similar, and hence does not result in distinct geographical differentiation of haplotypes in Peninsular Malaysia and Borneo Island.

In regions where the diversity of PfCSP Th2R/Th3R is low, a single allelic haplotype may predominate. This has been reported in Thailand [36, 45], Brazil [46] and Papua New Guinea [43], Vietnam, Indonesia and Myanmar [47]. In regions of high malaria transmission such as Africa, the diversity of PfCSP Th2R/Th3R is high and no distinct haplotypes seem to predominate [17, 21, 48]. On the other hand, in region of moderate of PfCSP Th2R/Th3R diversity, several haplotype may predominate. This is seen in Iran where three dominant haplotypes accounted for >90% of the 90 sequences studied [49]. Similar to the case of PfCSP in Iran, the moderate diversity of PkCSP Th2R/Th3R in our study is reflected by the predominance of several (five) haplotypes, with each giving rise to minor variants in different proportions (Fig 6).

## Conclusion

This study contributes to the understanding of the type and distribution of naturally occurring polymorphism in the *P*. *knowlesi* CSP gene. This study also provides a measurement of the genetic diversity of *P*. *knowlesi* and identifies the predominant haplotypes within Malaysia based on the C-terminal Th2R/Th3R regions.

## Supporting Information

S1 Fig Full amino acid sequence alignment of PkCSP from Peninsular Malaysia, Malaysia Borneo and Singapore. Amino acid residues identical to those of sequence KF861750 are indicated by dots. The nonrepeat and central repeat regions are indicated. The N-terminal nonrepeat region contains the conserved Region I (RI) KLKQP motif (blue) adjacent to the central repeat region. The C-terminal nonrepeat region contains a hypervariable region (yellow) flanking the C-terminal of the CR, the conserved Region II (RII, green) and Th2R (pink) and Th3R (pink) epitope sequences.
(XLSX)
